# Barriers to obesity management: a pilot study of primary care clinicians

**DOI:** 10.1186/1471-2296-7-35

**Published:** 2006-06-06

**Authors:** Valerie Forman-Hoffman, Amanda Little, Terry Wahls

**Affiliations:** 1 Center for Research in the Implementation of Innovative Strategies in Practice (CRIISP) Iowa City Veterans Affairs Medical Center, Iowa City, IA, USA; 2 Department of Internal Medicine, Carver College of Medicine, University of Iowa, Iowa City, IA, USA; 3 Carver College of Medicine, University of Iowa, Iowa City, IA, USA

## Abstract

**Background:**

Obesity is an increasing epidemic in both the US and veteran populations, yet it remains largely understudied in the Veteran's Health Administration (VHA) setting. The purpose of our study was to identify barriers to the effective management of obesity in VHA primary care settings.

**Methods:**

Three focus groups of clinicians from a Veteran's Affairs Medical Center (VAMC) and an affiliated Community Based Outpatient Center (CBOC) were conducted to identify potential barriers to obesity management. The focus groups and previously published studies then informed the creation of a 47-item survey that was then disseminated and completed by 55 primary care clinicians.

**Results:**

The focus groups identified provider, system, and patient barriers to obesity care. Lack of obesity training during medical school and residency was associated with lower rates of discussing diet and exercise with obese patients (p < 0.05). Clinicians who watched their own diets vigorously were more likely to calculate BMI for obese patients than other clinicians (42% vs. 13%, p < 0.05). Many barriers identified in previous studies (e.g., attitudes toward obese patients, lack of insurance payments for obesity care) were not prevalent barriers in the current study.

**Conclusion:**

Many VHA clinicians do not routinely provide weight management services for obese patients. The most prevalent barriers to obesity care were poor education during medical school and residency and the lack of information provided by the VHA to both clinicians and patients about available weight management services.

## Background

Obesity is an increasing epidemic in both the general US population [1] and veterans [2] and is an important etiologic factor in heart disease, diabetes, arthritis, depression, and various types of cancers [3,4]. It is estimated that among the 6 million users of Veterans Health Administration (VHA) services, 44% are overweight and an additional 25% are obese [5]. Although the U.S. Veterans Health Administration (VHA) is the largest integrated health care system in the United States [6], obesity in this setting has remained vastly understudied.

Despite numerous studies that have defined optimal body mass index (BMI) targets for patients, numerous provider-level barriers exist to the effective management of obesity in primary care. These barriers include lack of formal training of primary care practitioners in nutrition, obesity, and counseling on weight-related topics [7-12], perceived inability to change patient behaviors [12], lack of known effectiveness of treatments [8,13,14], negative attitudes toward obese patients [15-17], beliefs that patients are not interested or ready for treatment [9,12,19,20], and beliefs that obesity is the responsibility of the patient [21]. In addition, even though previous research has determined that patients have more confidence in weight counseling made by nonobese physicians, vegetarians, and those who used to be obese [20] and that nurse's weight impacts attitudes towards obesity and its treatment [19], it is unclear how provider's personal weight and exercise practices influence their obesity-related patient practices.

Even when providers do initiate health education and dietary counseling, there is still some controversy over whether these practices actually motivate patient behavior. Although previous studies have determined that the contextual framing of health behavior counseling in a positive (i.e., emphasizing the benefits of weight loss) versus negative (i.e., emphasizing the detriments of remaining obese) manner affects patient's receptivity [22,23], it is unknown whether framing style has an impact on the successful implementation of weight-related recommendations. Nonetheless, the manner by which clinicians discuss obesity with patients does affect patients' receptiveness to counseling [16], and even modest reductions in weight can lead to significant health benefits [24,25]. For example, it is estimated that a 10% reduction in weight can extend life expectancy an average of 2–7 months and can reduce lifetime medical expenditures of associated chronic conditions (e.g., heart disease, diabetes) by $2200–$5300 [26].

In addition to provider-related barriers, several previous studies have identified system-level barriers to obesity care. These include lack of payment by insurance companies for weight-related counseling and care [12,13,17], lack of time during patient visits [9,12,13], lack of available teaching materials for patients [9,12] and lack of infrastructure support/places to refer patients [27].

Although studies have been conducted to identify barriers to primary care management of obesity, it is unknown whether these barriers exist in the context of the VHA. For one, veterans have different characteristics than the general population (e.g., they are older, more likely to be male, possibly more resistant to change, possibly more functionally limited making exercise difficult) and the VHA system may not have the same barrier profiles as healthcare settings found in other sectors. The goal of this study was to explore obesity management practices in VHA primary health care. Specifically, we sought to identify provider- and system-level barriers to the effective management of obesity and to determine whether the identified barriers impacted obesity care in these settings. The identification of such barriers is an essential step in the development of more potent strategies for managing obesity and for decreasing the adverse effects of obesity in veterans.

The specific aims of the study were to:

• Describe weight management practices by VHA primary care clinicians

• Examine the relative importance of provider- and system-level barriers to the effective management of obesity

• Study whether barriers and/or personal weight management practices of clinicians impact care for obese patients

## Methods

Our aims were addressed by using focus groups to inform the creation and administration of a survey to primary care clinicians (attending physicians and physician assistants, resident physicians) in the Iowa City Veteran's Affairs Medical Center (VAMC) and primary care clinicians practicing at the Bettendorf Community Based Outpatient Clinic (CBOC). All research was approved by the University of Iowa Institutional Review Board (IRB) and the Veteran's Affairs (VA) Research and Development committees prior to initiation.

### Focus groups

Based on previous research, we developed a list of four open-ended focus group questions to ask participants. Three focus groups were conducted on 1) Iowa City VAMC attending clinicians (n = 3), 2) Iowa City resident physicians (n = 3), and 3) Bettendorf CBOC attending clinicians (n = 3). We asked each group of clinicians four open-ended questions about barriers to addressing and treating obesity, whether barriers were unique to the VA setting, and suggestions for improving current system of obesity care in the VA. Focus groups lasted approximately 45 minutes each, and responses were recorded by two members of the study team (VFH, AL). Themes emerging from the responses were compiled and combined into a master list of barriers identified (see Table 1).

**Table 1:** Obesity Care Barriers Identified During Focus Group Sessions

Lack of knowledge about VA weight management services and patient educational materials
Lack of time for patient counseling
Cynicism about effectiveness of obesity counseling and treatments
Lack of patient interest and readiness for change
Inadequate training in effective weight counseling, nutrition, behavioral modification
Lack of infrastructure support of weight-related referral services
Complex patients: more serious comorbid conditions demand majority of visit time and older patients may have functional limitations that prohibit physical activities

### Survey

Based on previous studies and focus group responses, we then created our 47-item survey. Questions addressed weight management practices for obese patients, both provider- and system-level barriers to weight management, beliefs about obesity and obesity care, usefulness of additional obesity-related services, provider personal weight management characteristics, context of weight framing (positive vs. negative), and demographic information. The context of the weight framing question is shown in Table 2. We included items on the clinicians' personal weight management practices to explore whether personal practices affected the likelihood of providing weight management services to obese patients. To test the face and content validity of the survey, we distributed it to a subset of three primary care clinicians to review its content and ease of use prior to the distribution of the survey to all eligible clinicians. We made several small changes to our survey to improve the flow of the questions.

**Table 2:** Framing of Weight Counseling Practices

When you counsel a patient on weight loss, which approach MOST resembles the one you generally use:
**Positive** "Mr. Jones, your weight is in the obese range. We need to try to get your weight down. Losing even a small amount of weight can help improve your risk for many diseases such as high blood pressure, heart disease, and arthritis. You also may feel better and have more energy."
**Negative** "Mr. Jones, your weight is in the obese range. We need to try to get your weight down. If you stay at this weight, you are at increased risk of diseases such as high blood pressure, heart disease, and arthritis. You're even likely to die early

### Participants

All primary care physician assistants (PAs) and physicians (attendings and residents) at the Iowa City VAMC and the Bettendorf CBOC were eligible to complete the study survey (n = 22 attendings and PAs and n = 75 residents).

### Survey administration

We administered the surveys in person to each of the three test groups (VAMC attending clinicians, VAMC resident physicians, CBOC attending clinicians) at normally scheduled meetings. The survey took 10–15 minutes to complete. We included a signature card with the survey to sign if they either a) did complete the survey or b) did not wish to complete the survey. This response card was collected separately from the surveys to protect anonymity and to facilitate follow-up of eligible providers who were present during the initial survey administration. We did not follow-up with anyone who returned the survey response card. To increase response rates, we followed-up with eligible clinicians who did not sign the signature card by sending the survey through email for completion. Our final sample consisted of 17 attending physicians and physician assistants (77% response rate) and 38 resident physicians (51% response rate).

### Data entry and analysis

Data was double-entered and imported into SPSS version 10 statistical software. We conducted simple descriptive statistics such as means and frequency tables for all variables. Body mass index (BMI) of the providers was calculated by dividing weight in kilograms by height in meters squared. We also recoded many of the likert scale variables into dichotomous variables to ease interpretation.

#### Weight management practices

- Always vs. Sometimes/Rarely/Never

We categorized responses to weight management practices into Always vs. Sometimes/Rarely/Never since nearly all respondents reported at least "Sometimes" performing these practices, and because guidelines suggest performing these practices *always *on overweight and obese patients.

#### Barriers, beliefs, and usefulness of additional services

- Strongly Agree/Agree vs. Neutral/Disagree/Strongly Disagree

We dichotomized in this manner since we wanted to compare affirmed responses to those with neutral or negative responses.

#### Provider body mass index (BMI)

- Overweight (BMI of 25+) vs. Not Overweight (BMI < 25)

We dichotomized in this manner based on the current definition for overweight (BMI of 25+).

#### Weight loss attempts

- Never vs. At Least Once or more frequent

We dichotomized in this manner so that we could distinguish never dieters from those who dieted at least once.

#### Dietary vigilance

- Moderately/Vigilantly/Very Vigilantly vs. Not at All/Slightly

We dichotomized in this manner given the distribution and interest in providers with at least moderate levels of dietary vigilance.

#### Exercise

- 3+ times per week vs. < 3 times per week

We dichotomized in this manner given our interest in providers with at least moderate levels of exercise, weekly.

We performed chi-square tests to assess the association between sets of two dichotomous variables of interest. For analyses where at least one expected cell size of the 2 × 2 table was less than 5, we used the Fisher's Exact Test to test for significance. We examined relationships between actual weight management practices and barriers, personal weight management practices of clinicians, framing of counseling practices, and demographic variables of clinicians. Barriers were identified as those items that most impacted rates of actual weight management practices.

## Results

Our sample was mostly comprised of males (63.6%), residents (69.1%), and clinicians from the Iowa City VAMC (85.5%). Over 40% of providers were overweight, most had tried to lose weight in the past (64.8%), most reported moderately to very vigilantly watching their diets (72.3%), and 29.6% reported exercising 3 or more times per week.

Clinician-reported rates of current weight management practices for obese patients were moderately low (Table 3). With respect to provider-level barriers to obesity care, few clinicians reported learning good obesity management practices in medical school (23.6%) and residency training programs (30.9%). Only 32.7% believed most obese patients are not ready to do anything about their weight, and few agreed (5.5%) that there are few effective treatments for obesity (Table 4). System-level barriers to obesity care included clinicians wanting more education about weight management services offered by the VA (94.4%), as well as recognition of the need for the VA to develop more comprehensive weight management services (83.6%) and to make obesity a higher priority (76.4%). Relatively few (27.3%) agreed that lack of payment by insurers hinders weight management practices in the VA healthcare system. Obesity-related beliefs included believing obesity to be a major public health problem and difficult to treat (98.2% and 96.4%, respectively) and considering obesity to be a disease (85.5%). Clinicians stated that older obese patients and those with comorbidity increase the likelihood of addressing and/or treating obesity. The need for more educational materials about weight management to pass out to patients (90.9%) and the need for an obesity educator (87.3%) and dietician (85.5%), physical therapist (81.8%) and behavioral counselor (83.6%) referrals for obese patients were expressed by most respondents. Most clinicians also believed that both group appointments for obese patients (72.7%) and having patients fill out readiness to change questionnaires about obesity prior to the visit (72.7%) would be helpful. Most respondents did not believe in patient monetary incentives for weight loss (44.4%) or that adding weight loss drugs to the formulary would lead to more frequent prescribing practices (25.5%). When asked to describe the context of their weight counseling messages, a majority of providers reported usually using positively framed messages to patients (77.8%) rather than negatively framed messages (22.2%).

**Table 3:** Current Weight Management Practices for Obese Patients

Among obese patients, how often do you...	Mean (sd)	n (%)	
Calculate Body Mass Index (BMI)	3.2 (0.8)	19 (35.2)	Always
		27 (50.0)	Sometimes
		5 (9.3)	Rarely
		3 (5.6)	Never
Inform patients of their BMI/weight	2.7 (0.7)	5 (9.3)	Always
		32 (59.3)	Sometimes
		13 (24.1)	Rarely
		4 (7.4)	Never
Recommend that the patient lose weight	3.3 (0.5)	19 (34.5)	Always
		34 (61.8)	Sometimes
		2 (3.6)	Rarely
		0	Never
Discuss diet with patients	3.1 (0.7)	15 (27.3)	Always
		32 (58.2)	Sometimes
		7 (12.7)	Rarely
		1 (1.8)	Never
Discuss exercise or physical activity levels	3.3 (0.6)	19 (34.5)	Always
		33 (60.0)	Sometimes
		3 (5.5)	Rarely
		0	Never
Refer patient to a dietician or nutritionist	2.7 (0.7)	4 (7.3)	Always
		31 (56.4)	Sometimes
		17 (30.9)	Rarely
		3 (5.5)	Never
Refer patient to physical therapy	2.3 (0.9)	4 (7.3)	Always
		20 (36.4)	Sometimes
		18 (32.7)	Rarely
		13 (23.6)	Never
Refer patient to a behavioral counselor	1.8 (0.8)	0	Always
		10 (18.2)	Sometimes
		21 (38.2)	Rarely
		24 (43.6)	Never
Refer patient to group weight loss treatment	1.7 (0.8)	0	Always
		9 (16.7)	Sometimes
		17 (31.5)	Rarely
		28 (51.9)	Never
Refer patient for bariatric surgery	1.5 (0.6)	0	Always
		4 (7.3)	Sometimes
		18 (32.7)	Rarely
		33 (60.0)	Never
Refer patient to inpatient weight	1.4 (0.6)	0	Always
loss therapy		3 (5.6)	Sometimes
		15 (27.8)	Rarely
		36 (66.7)	Never
Prescribe medications for weight loss	1.4 (0.6)	0	Always
		3 (5.5)	Sometimes
		16 (29.1)	Rarely
		36 (65.5)	Never

a Scale of 1 = never, 2 = rarely, 3 = sometimes, 4 = always

**Table 4:** Beliefs about Obesity and Weight Loss, Barriers to Care, and Usefulness of Additional Weight Management Services

	Mean (sd)^1^	n (%)	
**Provider Level Barriers**			
Most obese patients are not ready to do anything about their weight	3.1 (0.9)	0	Strongly Disagree
		16 (29.1)	Disagree
		21 (38.2)	Neutral
		14 (25.5)	Agree
		4 (7.3)	Strongly Agree
There is no evidence that physician-deliveredweight management counseling is effective	2.5 (0.8)	5 (9.1)	Strongly Disagree
		25 (45.5)	Disagree
		20 (36.4)	Neutral
		5 (9.1)	Agree
		0	Strongly Agree
There are no effective treatments for obesity	2.1 (0.8)	8 (14.5)	Strongly Disagree
		38 (69.1)	Disagree
		6 (10.9)	Neutral
		2 (3.6)	Agree
		1 (1.8)	Strongly Agree
I learned good obesity management practices in medical school	2.8 (1.0)	5 (9.1)	Strongly Disagree
		17 (30.9)	Disagree
		20 (36.4)	Neutral
		12 (21.8)	Agree
		1 (1.8)	Strongly Agree
I learned good obesity management practices during residency training	2.9 (1.0)	5 (9.1)	Strongly Disagree
		15 (27.3)	Disagree
		18 (32.7)	Neutral
		15 (27.3)	Agree
		2 (3.6)	Strongly Agree
I sometimes do not address obesity in fear of "ruining the relationship"	2.0 (0.8)	14 (25.5)	Strongly Disagree
		29 (52.7)	Disagree
		8 (14.5)	Neutral
		4 (7.3) 0	Agree Strongly Agree
			
**System-Level Barriers**			
I need more education about weight management services offered by the VA	4.1 (0.5)	0	Strongly Disagree
		0	Disagree
		3 (5.6)	Neutral
		41 (75.9)	Agree
		10 (18.5)	Strongly Agree
The VA needs more comprehensive weight management services	3.9 (0.6)	0	Strongly Disagree
		2 (3.6)	Disagree
		7 (12.7)	Neutral
		39 (70.9)	Agree
		7 (12.7)	Strongly Agree
The VA needs to make obesity a higher priority	3.8 (0.8)	0	Strongly Disagree
		4 (7.3)	Disagree
		9 (16.4)	Neutral
		35 (63.6)	Agree
		7 (12.7)	Strongly Agree
I would be more likely to address obesity with patients if visit times were longer	3.5 (1.0)	3 (5.5)	Strongly Disagree
		6 (10.9)	Disagree
		12 (21.8)	Neutral
		28 (50.9)	Agree
		6 (10.9)	Strongly Agree
Lack of payment by insurers hinders my weight management practices in VA primary care	2.8 (1.0)	5 (9.1)	Strongly
		16 (29.1)	Disagree
		19 (34.5)	Neutral
		19 (27.3)	Agree
		0	Strongly Agree
			
**Beliefs about Obesity and Weight Loss**			
Obesity is a very important public health problem	4.8 (0.5)	0	Strongly Disagree
		0	Disagree
		1 (1.8)	Neutral
		9 (16.4)	Agree
		45 (81.8)	Strongly Agree
Obesity is difficult to treat	4.4 (0.6)	0	Strongly Disagree
		0	Disagree
		2 (3.6)	Neutral
		27 (49.1)	Agree
		26 (47.3)	Strongly Agree
Obesity is a disease	4.2 (0.9)	1 (1.8)	Strongly Disagree
		1 (1.8)	Disagree
		6 (10.9)	Neutral
		23 (41.8)	Agree
		24 (43.6)	Strongly Agree
I am more likely to address obesity if the patient is younger	3.0 (1.0)	1 (1.8)	Strongly Disagree
		22 (40.0)	Disagree
		14 (25.5)	Neutral
		15 (27.3)	Agree
		3 (5.5)	Strongly Agree
Most VA patients attribute their obesity to an external cause (e.g., agent orange) rather than an internal cause (e.g., their lack of self discipline	2.6 (0.8)	1 (1.8)	Strongly Disagree
		27 (49.1)	Disagree
		19 (34.5)	Neutral
		5 (9.1)	Agree
		2 (3.6)	Strongly Agree
Having multiple comorbidities (e.g., diabetes, hypertension, osteoarthritis) makes it *less *likely that I will address obesity	1.9 (1.1)	26 (47.3)	Strongly Disagree
		19 (34.5)	Disagree
		3 (5.5)	Neutral
		6 (10.9)	Agree
		1 (1.8)	Strongly Agree
			
**Usefulness of Additional Services**			
The VA needs to develop educational materials about weight management to pass out to patients	4.1 (0.6)	0	Strongly Disagree
		1 (1.8)	Disagree
		4 (7.3)	Neutral
		39 (70.9)	Agree
		11 (20.0)	Strongly Agree
Having an obesity educator in the VA would be helpful	4.1 (0.8)	1 (1.8)	Strongly Disagree
		1 (1.8) 5 (9.1)	Disagree Neutral
		33 (60.0)	Agree
		15 (27.3)	Strongly Agree
Having a referral box for a dietician on CPRS would be helpful	4.0 (0.8)	2 (3.6)	Strongly Disagree
		0	Disagree
		6 (10.9)	Neutral
		38 (69.1)	Agree
		9 (16.4)	Strongly Agree
Having a referral box for a physical therapist on CPRS would be helpful	3.9 (0.8)	2 (3.6)	Strongly Disagree
		0	Disagree
		8 (14.5)	Neutral
		39 (70.9)	Agree
		6 (10.9)	Strongly Agree
Having a referral box for a behavioral counselor on CPRS would be helpful	3.9 (0.8)	2 (3.6)	Strongly Disagree
		1 (1.8)	Disagree
		6 (10.9)	Neutral
		39 (70.9)	Agree
		7 (12.7)	Strongly Agree
Group appointments for obesity (e.g., nutrition class, exercise class, behavior change class) would be helpful	3.9 (0.9)	1 (1.8)	Strongly Disagree
		4 (7.3)	Disagree
		10 (18.2)	Neutral
		26 (47.3)	Agree
		14 (25.5)	Strongly Agree
Having patients fill out a readiness to change questionnaire about obesity prior to the visit would be helpful	3.7 (0.9)	2 (3.6)	Strongly Disagree
		3 (5.5)	Disagree
		10 (18.2)	Neutral
		35 (63.6)	Agree
		5 (9.1)	Strongly Agree
The VA should give monetary incentives for weight loss (e.g. cash, reduction of copays, free obesity-related services)	3.1 (1.3)	8 (14.8)	Strongly Disagree
		12 (22.2)	Disagree
		10 (18.5)	Neutral
		15 (27.8)	Agree
		9 (16.7)	Strongly Agree
If weight loss drugs were on formulary, I would prescribe them more frequently	2.7 (1.0)	7 (12.7)	Strongly Disagree
		18 (32.7)	Disagree
		16 (29.1)	Neutral
		14 (25.5)	Agree
		0	Strongly Agree

^1 ^Scale of 1 = strongly disagree, 2 = disagree, 3 = neutral, 4 = agree, 5 = strongly agree

The barrier that most greatly impacted discussing diet or exercise with patients was lack of learning good obesity practices during medical school and residency training programs (Table 5). Clinicians who learned good obesity practices in medical school were more likely to discuss diet or exercise with obese patients than those who reported that they did not learn good obesity practices in medical school (62% vs. 31%, respectively, p < 0.05). Likewise, clinicians who learned good obesity practices in residency training programs were more likely to always discuss diet or exercise with obese patients than those who reported that they did not learn good obesity practices in residency training programs (59% vs. 29%, respectively, p < 0.05). The vigilance with which clinicians reported watching their own diets was significantly associated with the likelihood of calculating the BMI of their obese patients. Whereas 42% of clinicians who moderately to very vigilantly watched their diets reported always calculating BMIs of obese patients, only 13% of providers who less vigilantly watched their diets did so.

**Table 5:** Current Weight Management Practices for Obese Patients Associated with Barriers, Beliefs, Personal Weight-Related and Clinical Characteristics

	For obese patients, percent of providers who always				
	
Barriers, Beliefs, Personal Weight-Related and Clinical Characteristics	Calculate BMI	Recommend weight loss	Discuss diet or exercise	Refer for wt. services	Counsel in positive context
Learned good obesity practices in medical school					
Agree	39	54	62	23	67
Not agree	34	29*	31**	5^*-f^	81
Learned good obesity practices in residency					
Agree	44	53	59	19	81
Not agree	32	26*	29**	5	76
Obesity is a disease					
Agree	35	34	40	9	83
Not agree	38	38	25	13	50^*-f^
Training level					
Attending physician/PA	44	29	41	13	94
Resident physician	32	37	37	8	70^*-f^
Own BMI					
Not overweight (<25)	39	31	44	16	78
Overweight (25+)	27	41	32	0^*-f^	76
Watch own diet					
Moderately-Very Vigorously	42	36	39	11	79
Not at all- Slightly Vigorously	13**	33	40	7	73
Own exercise practices					
3+ times per week	50	31	44	6	100
<3 times per week	27*	37	37	11	68^*-f^

*p < 0.10 based on Chi-Square statistic ** p < 0.05 based on Chi-Square statistic ^f ^Fisher's Exact Test

With respect to whether clinicians reported typically counseling obese patients in a positive or negative context, responses to this item differed (nearly significantly) by one obesity belief item and one clinical characteristic. While 83% of clinicians who agreed that obesity is a disease counseled obese patients in a positive context, only 50% of clinicians who did not agree that obesity is a disease counseled obese patients in a positive context (p < 0.10). Likewise, 94% of attending clinicians reported counseling obese patients positively while 70% of residents reported counseling obese patients positively (p < 0.10).

## Discussion

Our study shows that many primary care clinicians in the VA do not regularly counsel and refer obese patients to weight management services. The barrier most strongly related to providing diet and exercise counseling was poor obesity education during medical school and residency training programs. In fact, less than a third of respondents reported that they had learned good obesity management practices during medical school and residency training programs. This finding of poor nutrition, exercise, and behavioral counseling in medical and residency training programs has been corroborated in a number of previous studies [7,8,12]. In our study, personal dietary vigilance by clinicians also impacted the likelihood of regularly calculating the BMI of obese patients, with more vigilant clinicians being more likely to calculate BMI. Previous studies, too, have found similar results in that physicians with a personal history of obesity or vegetarianism were more likely to provide nutrition and weight counseling to patients [28]. Another study by Hash et al. [20] concluded that patients were more receptive to health counseling given by nonobese physicians.

Contrary to previous studies [17,29], our study did not find that clinicians feel that their weight loss efforts are futile. Our surveyed clinicians largely believed that many obese patients are ready for weight loss, that physician-delivered weight counseling can be successful, and that effective weight management tools are available.

Although a number of barriers identified in previous studies were confirmed in the current study, several barriers such as attitudes toward obese patients and lack of insurance payments for obesity care were not related to actual weight management practices. Insurance coverage may not have been a barrier since most veterans receiving care in the VHA have coverage for a wide array of services, including obesity-related services, unlike many patients found in the general population.

In addition, clinicians reported access to educational materials for obese patients, ability to refer patients to an obesity educator, dietician, physical therapist, behavioral counselor, and group obesity appointments, and use of a readiness to change questionnaire about weight loss for obese patients as useful weight management services for the VA to implement. Many of these services will be offered as the VHA's Managing Overweight and Obesity in Veterans Everywhere (MOVE!) Program is nationally rolled-out. The program is multidisciplinary in nature and includes five steps of care ranging from patient education to bariatric surgery provided by dieticians, behavioral counselors, obesity educators, clinicians, nurses, physical therapists, and surgeons.

Our study found that the context of weight management counseling practices vary, and that these practices differ by beliefs about obesity and by training level. Clinicians who believe obesity is a disease are more likely to counsel obese patients in a positive context than clinicians who do not believe obesity to be a disease. This may imply that clinicians who believe obesity to be a real clinical entity would be more compassionate towards obese patients than clinicians who feel that obesity is a character flaw or linked to lack of willpower. This finding supports that of Scott et al. [30], who found that clinicians who were the most likely to counsel patients on weight management initiated conversations by "medicalizing" the obesity. Our results also showed that attending clinicians were more likely than resident physicians to counsel positively. Whether this finding is due to the years of experience of the attending physicians or to a shift in obesity counseling norms is indeterminable by this study. In addition, our sample sizes were very small in these analyses, and most context of counseling items required Fisher's Exact Tests for 2 × 2 analyses given the small cell sizes for several of the cells.

Other limitations of our study include our small response rate of resident physicians, our exclusion of nurses, and our sample of only one VAMC and one CBOC. This project served to pilot test our survey, which we plan to distribute to more VAMCs and CBOCs in the near future. We note that the generalizability of our findings may be limited to our region and therefore, should be interpreted with caution. In addition, our pilot study resources prohibited our study of nurses, although future studies are planned to identify obesity-related practices and barriers that nurses in the VA healthcare system face, given their important role in obesity management. High response rates from resident physicians are difficult to capture given their busy schedules and demands from other research projects. We recognize, however, that the residents most likely to have responded may be more conscientious than non-responders, and therefore the rates of various obesity practices may be over-inflated in this study. Nevertheless, our rates of most practices were sufficiently low to warrant additional attention to obesity management in the VA healthcare system. Lastly, due to time constraints, we were not able to perform all of the appropriate reliability testing on our survey instrument, such as test-retest reliability. We are currently conducting these tests and will have completed them before our next round of survey administration.

Our study does, however, have several strengths. For one, this is the first time primary care management of obesity has been examined empirically in the VA to our knowledge. Secondly, although we only gathered respondents from two settings, we did administer the survey to one large VAMC and one smaller CBOC, and also had respondents with two different training levels (attendings and residents). This mix of providers will help increase the reliability of our findings.

## Conclusion

Our initial results suggest that efforts to impact the current epidemic of obesity in veterans focus on interventions to improve clinicians' knowledge of obesity management and nutrition and to increase awareness of available obesity management services. This research is necessary to understand how VHA can improve the quality of obesity management. This focus on quality of care and evidence-based practices has accompanied the VHA's shift from a system of tertiary/specialty and inpatient-based care to a primary, outpatient-based care [31, 32].

As the VA rolls out the MOVE! initiative, we can use the results of our study to modify the program appropriately in response to the identified provider-level and system-level barriers. Additionally, our pilot study findings will inform future multi-site provider surveys administered across multiple VAMCs. In addition, given the broad effects of obesity, the results of this study are relevant to many of the other initiatives developed by the VA to improve the control of blood pressure, serum lipids, and blood glucose in an effort to decrease cardiovascular morbidity and mortality, as well as to other healthcare settings incorporating evidence-based care practices into their quality improvement initiatives.

## Competing interests

The author(s) declare that they have no competing interests.

## Authors' contributions

VFH designed the study, performed the statistical analyses, and drafted the manuscript. AL assisted with the focus groups and study coordination, helped performed the literature search, and helped with manuscript drafting. TW assisted with the conceptualization of the study design, as well as reviewing and revising manuscript drafts.

## Pre-publication history

The pre-publication history for this paper can be accessed here:


